# Changes in Elements and Relationships among Elements in Intervertebral Disc Degeneration

**DOI:** 10.3390/ijerph19159042

**Published:** 2022-07-25

**Authors:** Rafał Staszkiewicz, Kamil Bryś, Dorian Gładysz, Marcin Gralewski, Michał Garczarek, Marcin Gadzieliński, Jerzy Wieczorek, Wiesław Marcol, Aleksander Ostenda, Beniamin Oskar Grabarek

**Affiliations:** 1 Department of Neurosurgery, 5th Military Clinical Hospital with the SP ZOZ Polyclinic in Krakow, 30-901 Krakow, Poland; gladyszdorian875@gmail.com (D.G.); marcingralewski79@gmail.com (M.G.); michal.people@wp.pl (M.G.); marcin.gadzielinski@gmail.com (M.G.); bgrabarek7@gmail.com (B.O.G.); 2 Department of Histology, Cytophysiology and Embryology, Faculty of Medicine in Zabrze, Academy of Silesia in Katowice, 41-800 Zabrze, Poland; brysek12@gmail.com; 3 Department of Agricultural and Environmental Chemistry, University of Agriculture in Krakow, 31-120 Krakow, Poland; jerzy.wieczorek@urk.edu.pl; 4 Department of Physiology, School of Medicine in Katowice, Medical University of Silesia, 40-752 Katowice, Poland; wmarcol@tlen.pl; 5 Department of Neurosurgery, Provincial Specialist Hospital No. 2 in Jastrzębie-Zdrój, 44-300 Jastrzębie-Zdrój, Poland; 6 Faculty of Medicine in Zabrze, Academy of Silesia in Katowice, 41-800 Zabrze, Poland; aleksander.ostenda@wst.com.pl

**Keywords:** intervertebral disc degeneration, Pfirrmann scale, VAS, microelements, spine

## Abstract

Intervertebral disc degeneration (IVDD) is a complex and progressive process of disc aging. One of the most important causes of changes in the internal environment, leading to IVDD, can be changes in the concentration of individual metal elements. This study aimed to analyze the concentrations of copper, iron, manganese, lead, zinc, sodium, potassium, phosphorus, and calcium in the degenerated intervertebral discs of the lumbosacral spine, compared to healthy intervertebral discs. The study group (S) consisted of 113 Caucasian patients, qualified by a specialist surgeon for IVDD of the lumbosacral spine. The control group (C) consisted of 81 individuals. The biological material was obtained from Caucasian human cadavers during post-mortem examination. The concentrations of individual elements were assessed using inductively coupled plasma–optical emission spectroscopy (ICP-OES). Statistically significant differences in the concentrations of microelements, depending on the degree of pain intensity, were noted for only potassium (*p* < 0.05). Statistically significant differences in the concentrations of the assessed microelements, depending on the degree of radiological advancement of the lesions, were noted for copper and iron (*p* < 0.05). In the degenerated intervertebral discs, the strongest relationships were noted between the concentrations of zinc and lead (*r* = 0.67; *p* < 0.05), zinc and phosphorus (*r* = 0.74; *p* < 0.05), and zinc and calcium (*r* = 0.77; *p* < 0.05). It has been indicated that, above all, the concentrations of copper and iron depend on the advancement of radiological changes, according to the Pfirrmann scale; however, no influence on the pain intensity, depending on the concentration of the assessed elements, was found.

## 1. Introduction

The intervertebral disc (IVD) is an anatomical structure with three compartments: a centrally located nucleus pulposus (NP), surrounded by the annulus fibrosus (AF), and the cartilaginous endplate (CE). It is known that 70–90% of the NP consists of water and has a pasty consistency. It consists of a relatively small number of chondrocytes and a network of collagen fibers, mainly type II, which constitute 20–25% of the NP dry matter, suspended in an aqueous solution that supports the proteoglycan fibers. On the other hand, AF is a compact structure in which type I and II collagen fibers alternate, with type I collagen predominating in the outer parts of AF, forming 15–25 separate, concentrically arranged layers with an oblique course from one adjacent vertebral body to another. The described structure of the AF allows for the preservation of the semi-liquid NPs within the IVD, ensuring flexibility and multi-axis movement, maintaining the correct structure of the IVD, especially with high axial loads in the IVD. The physiological components of a normal IVD are in a state of mutual homeostasis [1,2,3].

The degenerative process of IVD of the lumbosacral spine results from repeated overload and increased spine mobility. Of course, the factors that predispose an individual to the occurrence of IVD include age, but also, for example, scoliosis or trauma within a given section of the spine. In addition, the degeneration process of IVD is closely related to its dehydration, which results in the formation of cracks within the NP and the creation of a “vacuum”. There are four main stages of herniation of the NP: (1) disc bulging (protrusion); (2) protruded nucleus pulposus; (3) herniated nucleus pulposus (extrusion); (4) prolapse of the nucleus pulposus (sequestration, free fragment). The degeneration of the IVD is accompanied by a cascade of changes in the individual components of the IVD [4,5].

A healthy IVD is characterized by very poor vascularization and vascularity. The IVD metabolism is based on the passive transport of nutrients and water through its border laminae. The IVD blood supply in the period from development to the first year of life takes place mainly through the branches of the spinal artery. In the following years, most of these vessels disappear. Only the anterior and posterior longitudinal ligaments remain supplied with blood, as well as very superficial outer layers of the fibrous ring, up to a depth of approximately 3.5 mm into the IVD. Tiny branches from the segmental arteries supply blood to the vertebral bodies and IVD from the vertebral bodies. The vessels supplying blood to the outer parts of the IVD are accompanied by nerve fibers, which are branches of the retrograde sinus–spinal nerve leading from the dorsal part of the spinal nerve root [6,7,8,9].

As a result of the progressive degenerative process, the pH of the matrix decreases, which results in the following: destruction of proteoglycans; activation of nociceptive vanillin receptors sensitive in an acidic environment; and increased secretion of inflammatory mediators, namely interleukin 6 (IL-6), interleukin 8 (IL-8), platelet activating factor, and calcitonin gene-derivative peptide [10,11,12,13].

The above-mentioned processes, which take place under the influence of the pH reduction, lead to the formation of new nerve endings and sensitization of the present nerves, due to the increased concentration of neutrophins, leading to the occurrence of pain [8,9,14].

The degenerative disease manifests itself at an early age. Approximately 20% of adolescents have mild symptoms of the disease. With age, the risk of disc degeneration increases. Urban et al. noticed that in a group of 70-year-olds, 60% of the IVD was severely degenerated [15].

One of the most important causes of changes in the internal environment leading to IVDD can be changes in the concentration of individual metal elements. Performing a quantitative analysis of the elements present in the intervertebral disc, in which degeneration occurs, enables the identification of the metals that may significantly affect the progression of degenerative changes, or the regeneration of the damaged intervertebral disc [16].

The aim of this study was the assessment of the concentrations of copper (Cu), iron (Fe), manganese (Mn), lead (Pb), zinc (Zn), sodium (Na), potassium (K), phosphorus (P), and calcium (Ca) in the IVDD of the lumbosacral spine, compared to healthy IVD.

## 2. Materials and Methods

### 2.1. Ethics

This study was performed in accordance with the guidelines of the 2013 Declaration of Helsinki on human experimentation. Data confidentiality and patient anonymity were maintained at all times. Informed consent was obtained from all of the patients. The approval of the Bioethical Committee operating at the Regional Medical Chamber in Krakow, no. 162/KBL/OIL/2021, 11 June 2021, was obtained for this study.

### 2.2. Study Group

The study group (S) consisted of 113 Caucasian patients (55 women and 58 men; 45.5 ± 1.5 years), qualified by a specialist surgeon for degeneration of the IVD of the lumbosacral spine. In the study group, normal weight, as determined by the value of Body Mass Index (BMI), was observed in 54 patients (BMI 18.5–24.9), including 37 women and 17 men; overweight (BMI 25–29.9) in 42 patients, including 17 women and 15 men, and obesity (BMI > 30) in 17 patients, including 1 woman and 26 men.

All of the patients underwent a complete neurological examination and Magnetic Resonance Imaging (MRI) of the lumbosacral spine (1.5 T), conducted during T2, before the surgery. The degree of the radiological advancement of the degenerative changes in the intervertebral disc was assessed, using Pfirrmann’s scale. In the question examination, none of the patients had any knowledge of being inadvertently exposed to heavy metal pollution. Additionally, the patients were asked to rate their pain intensity on a scale from 0 to 10 points, where 0 indicated no pain and 10 indicated significant intensification of pain. The inclusion and exclusion criteria are shown in Table 1.

### 2.3. Control Group

The control group (C) consisted of 81 individuals (43 women and 38 men; 31.5 ± 1.5 years). The biological material was obtained from Caucasian human cadavers, during post-mortem examination, which was conducted no more than 48 h from the moment of death and after the death certificate was obtained. The clinical data were collected, based on a detailed analysis of the available medical documentation and an interview with immediate family members. In the question examination, none of the patients had any knowledge of being inadvertently exposed to heavy metal pollution. In the control group, normal weight (BMI 18.5–24.9) was observed in 23 individuals, including 16 women and 7 men; overweight (BMI 25–29.9) in 40 individuals, including 24 women and 16 men; and obesity (BMI > 30) in 18 individuals, including 3 women and 15 men. The inclusion and exclusion criteria are summarized in Table 2.

#### Hematoxylin and Eosin Staining of IVD Obtained from the Control Group

In order to exclude the degenerative changes in IVD collected from the control group, staining of the obtained material was performed using Hematoxylin and Eosin dyes (H&E staining). The specimens for histological evaluation were fixed in 10% formalin (Sigma Aldrich, St. Louis, MO, USA, catalog number HT501128) for 24 h, then dehydrated and embedded in paraffin and cut into 5 μm thick sections, and then stained with a Hematoxylin and Eosin staining kit (Abcam, Abcam, Cambridge, MA, USA, catalog number ab245880), in accordance with the manufacturer’s protocol.

### 2.4. Determination of the Content of Elements in Intervertebral Discs of the Lumbosacral Spine

In the analyzed intervertebral discs, the content of the selected elements was determined using inductively coupled plasma–optical emission spectrometers (ICP-OES), following prior mineralization of the analyzed material, using the “wet” method in a closed system.

The dry matter content samples of 0.3–0.5 g were digested in a mixture of concentrated 6 cm^3^ of 65% acids—nitric (V) HNO_3_ (Sigma Aldrich, St. Louis, MO, USA, catalog number 7697-32-2) and 1 cm^3^ of 30% hydrochloric acid (Sigma Aldrich, St. Louis, MO, USA, catalog number 7647-01-0) —with Merck suprapur high purity salts, using the Anton Paar Multiwave 3000 microwave oven. The mineralization was conducted in Teflon containers at maximum power for the device (1400 W) for 25 min (including the time taken to reach maximum power—10 min; time to maintain power level—15 min).

After mineralization, the samples were quantitatively filtered into 10 cm^3^ volumetric flasks with HNO_3_ solution. In such prepared solutions, the content of the selected elements was determined using the Optima 7300 Dual View atomic emission spectrometer (Perkin Elmer Polska, Warszawa, Poland). All of the samples were analyzed in triplicate.

### 2.5. Statistical Analysis

The STATISTICA 13 PL software (Cracow, Poland) was used to perform the statistical analysis. In the first step of the analysis, the results were evaluated in terms of normal distribution, using the Shapiro–Wilk test. Due to the fulfillment of the test assumptions, further statistical analyses were conducted using parametric methods—either the Student’s *t*-test for comparing two independent groups (degenerated intervertebral discs vs. healthy intervertebral discs) or, to compare three or more groups, the ANOVA variance analysis followed by Tukey’s post-hoc test (*p* < 0.05). The post-hoc Tukey’s test was only performed when the *p*-value of one-way ANOVA variance was less than 0.05 (*p* < 0.05). Additionally, Pearson correlation analysis was performed between the concentrations of selected microelements (*p* < 0.05).

## 3. Results

### 3.1. H&E Staining for Healthy Interverbal Disc

**Figure 1:**
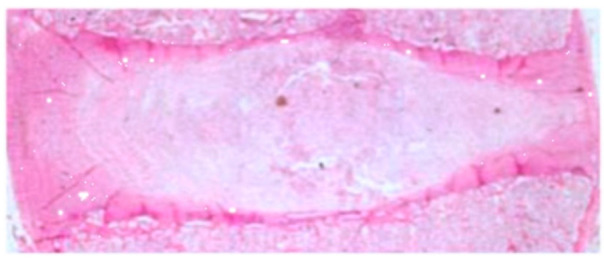
Hematoxylin and eosin (H&E)-stained images under low magnification (×50) (own drawing).

Based on the histological evaluation of the IVD preparation obtained from the control group, no degenerative changes were found. In Figure 1, we present an example of the histology examination of an IVD obtained from the control group (H&E staining).

### 3.2. Concentration of Selected Elements in Degenerated and Healthy Intervertebral Discs

Based on the conducted analysis of the concentrations of the elements, it could be observed that, regardless of whether the intervertebral disc was degenerated or not, the highest concentrations were found for iron, zinc, and copper. In the healthy intervertebral discs, higher concentrations were found for copper, sodium, potassium, and phosphorus, compared to the intervertebral discs affected by the degenerative process. In the case of the remaining elements, a different direction of change was noted. Statistically significant differences in the concentrations of the elements in the degenerated intervertebral discs, compared to the healthy intervertebral discs, were recorded for copper, magnesium, potassium, and calcium (*p* < 0.05; Table 3). For the other elements, the changes were not statistically significant (*p* > 0.05; Table 3).

In addition, we did not observe statistically significant changes in any of the concentrations of the elements among the sexes in both the study and control groups (Table 4; Student’s *t*-test; *p* > 0.05).

Therefore, we also did not notice statistically significant differences in the concentrations of the micronutrients assessed in a given group (healthy or tested), when we adopted the BMI value (one-way ANOVA test; *p* > 0.05) as the criterion. However, for most of the elements, statistically significant differences in the concentrations of micronutrients were found for the comparison between the study group and the control group within the same BMI value (Table 5; Student’s *t*-test; *p* < 0.05).

### 3.3. Concentrations of Selected Elements in Degenerated Intervertebral Discs, Depending on Pain Intensity

In the second stage, changes in the concentrations of the assessed elements were assessed depending on pain intensity, according to the Visual Analogue Scale (VAS scale). No patients declared a pain intensity level of 0 or 1. The pain intensity among the patients was as follows: 2 points—2 patients; 3 points—6 patients; 4 points—15 patients; 5 points—11 patients; 6 points—30 patients; 7 patients each assessed their pain intensity as 7 and 9 points; 8 points—26 patients; and 10 points—10 patients.

The analysis did not include the results for the pain intensity of 2 according to the VAS scale, due to its being too small a group. We did not observe statistically significant differences in the concentration of the microelements depending on pain intensity (Table 6; *p* > 0.05).

### 3.4. Concentrations of Selected Elements in Degenerated Intervertebral Discs, Depending on the Degree of Degeneration, According to the Pfirrmann Scale

In the next stage, the changes in the concentrations of the selected elements were assessed depending on the advancement of the degenerative changes in the intervertebral discs, according to the Pfirrmann scale. In total, 27 patients displayed stage 2 radiological lesion advancement; 43 patients displayed stage 3; 32 patients displayed stage 4; and 11 patients displayed stage 5. Statistically significant differences in the concentrations of the assessed microelements, depending on the degree of the radiological lesions’ advancement, were noted for the following: copper (*p* = 0.014; one-way analysis of variance, ANOVA), between grade 2 and 4 (post-hoc Tukey’s test; *p* = 0.017), and grade 2 and 5 (post-hoc Tukey’s test; *p* = 0.033); iron (post-hoc Tukey’s test; *p* = 0.003), between grade 2 and 3 (post-hoc Tukey’s test; *p* = 0.011), grade 2 and 4 (post-hoc Tukey’s test; *p* = 0.002), and grade 3 and 4 (post-hoc Tukey’s test; *p* = 0.027). For the other elements, their concentrations, depending on the degree of advancement of the changes, according to the Pfirrmann scale, were not statistically significant (*p* > 0.05; Table 7).

### 3.5. Correlation between the Concentrations of Individual Microelements in Healthy and Degenerated Intervertebral Discs

In the final stage, we determined the relationships between the concentrations of the individual microelements in the degenerated (Table 8; *p* < 0.05) and healthy (Table 6; *p*< 0.05) intervertebral discs. In the degenerated intervertebral discs, the strongest relationships were noted between the concentrations of zinc and lead (*r* = 0.67; *p* < 0.05), zinc and phosphorus (*r* = 0.74; *p* < 0.05), and zinc and calcium (*r* = 0.77; *p* < 0.05).

In turn, in the intervertebral discs unaffected by the degenerative process, stronger relationships between the individual elements were noted. The strongest significant relationships were found between the concentrations of magnesium and sodium (*r* = 0.90; *p* < 0.05), and of calcium and ferrum (*r* = 0.70; *p* < 0.05). The results of the correlation analysis between the concentrations of the individual elements in the healthy intervertebral discs are presented in Table 9.

## 4. Discussion

The quantitative analysis of the content of trace elements in the human intervertebral discs affected by the degenerative process is of significant importance [16]. It should be remembered that some of the elements, such as copper, zinc, and magnesium, participate in the metabolic processes, while others, such as lead and cadmium, provide information about exposure to environmental pollutants and have accumulative properties in the examined tissue. In most of the studies, the concentration of the elements was determined in the bone tissue because it shows the ability to accumulate; thus, the obtained results reflect the content and their change in the entire body. In turn, the concentration of the elements in the serum, urine, or cerebrospinal fluid corresponds to the periodic reaction of the body [17,18,19].

Nowakowski et al. confirmed, in all 18 of their IVDD samples, the concentrations of Al, Cu, Mg, and Zn. Furthermore, the positive correlation between age and the content of the toxic element, aluminum, was determined in the intervertebral discs affected by the degenerative process [20].

The observations of Hangai et al., who showed that systemic factors significantly influence the metabolism of IVD and its degeneration, are interesting. These authors, assessing a population of 170 patients aged 51–86 years, confirmed that age (Odds Ratio; OR 2.14–3.05), high BMI (OR 2.98–3.58), physical work (OR 3.34), lack of physical activity (OR 3.35), and a high concentration of low-density lipoprotein cholesterol (OR 2.65) significantly contribute to the development of IVD degenerative disease, of the lumbosacral (L/S) section of the spine. This indicates that both environmental factors, such as air pollution, diet, stimulants, and medications used, as well as lifestyle and diet, have a significant indirect impact on the occurrence and progression of IVD, of the L/S section of the spine, as suggested by our research on the determination of the concentrations of the elements in patients with normal body weight, overweight, and obesity [21]. Of course, other studies also show that these factors contribute to the degeneration of the IVD [22,23,24].

To the best of the authors’ knowledge, the concentrations of the selected micronutrients in the individual IVD compartments have not been described so far. However, it should be assumed that the highest concentration of elements would be found within the CE, because this structure is composed of vitreous cartilage and plays the role of the CE, which is a thin layer of hyaline cartilage positioned between the vertebral endplate and NP that functions both as a mechanical barrier and as a gateway for nutrient transport into the disc. In turn, the lowest concentration of elements most likely occurs in the NP, as it consists of 90% water and the amount of chondrocytes is very small [1,2,3]. In addition, taking into account the etiology of the IVD degeneration related to IVD dehydration, a higher concentration of micronutrients, including sodium and potassium ions, in the CE of the degenerated IVD increases the osmotic power of absorption and allows the CE to retain water, even in the degenerated IVDs [25]. The lack of differences in the individual compartments described in the literature is most likely due to the fact that, in the study group, the IVD is collected using the en bloc method, which essentially prevents the identification of NP and AF. Only in the case of IVD obtained from the control group would it be possible to isolate the IVD compartments, because, during the autopsy, after the donor qualification, the front surface of the L/S spine is exposed in a manner typical of post-mortem examinations, and then, using a flat dissection knife, it is completely cut out, allowing the appropriate IVD of the L/S spine to be obtained.

Based on our analysis, it was indicated that the most important elements relating to the IVD are copper, magnesium, potassium, and calcium (*p* < 0.05). On the other hand, the potential utility in differentiating the degree of advancement in the degenerative changes in the intervertebral disc was determined for the changes in the copper and iron concentrations (*p* < 0.05).

### 4.1. Cooper

The changes in the copper concentration observed by us are in contradiction to those noted by Kubaszewski et al. [26]. However, these authors conducted their studies on a group of 22 patients, who demonstrated second- and third-degree radiological degeneration of the spine. Additionally, Dąbrowski et al. found a higher concentration of copper in the degenerated hip joints compared to the healthy ones [27], which could be the result of the greater accumulation of elements in the bone tissue than in the intervertebral discs. One of the potential reasons for us noting a lower copper concentration in the degenerated intervertebral discs compared to the healthy ones is the fact that the group of patients in the study group was treated for a long time pharmacologically, using non-steroidal anti-inflammatory drugs and rehabilitation, which could lead to a reduction in the lipid peroxidation and systemic oxidative stress [28]. It cannot be ruled out that the collection of copper concentration results, other than those previously recorded in degenerated intervertebral discs by Kubaszewski et al. [26], and inconsistent with the observations of Mahmood et al. [29], who indicated a higher concentration of copper in patients with knee osteoarthritis, depending on age and the duration of the disease, may be related to the fact that we assessed the concentration of elements in material that was not composed of bone or cartilage tissue [29]. We also did not indicate a statistically significant relationship between the concentrations of zinc and copper.

### 4.2. Magnesium

The second most important element in the degeneration process of the intervertebral disc is magnesium, with a higher concentration determined in the degenerated intervertebral discs. The changes in its concentration, however, did not depend on the advancement of the changes (*p* > 0.05). Tohno et al. found a mean concentration of calcium of 1.196 mg/g^−1^, in the degenerated intervertebral disc samples obtained from nine deceased patients, which is significantly lower than in our study, which may result from both the smaller study group and the collection of intervertebral discs from human corpses. Moreover, information about the degree of degeneration and its etiology was not included [30]. In turn, Yamada et al. [31] observed a magnesium concentration of 445 mg·kg^−^^1^, and Jurkiewicz et al. [32] found the concentration of magnesium in the bone to be 1883.5 µg/g, which is less than in the intervertebral discs in our analysis, regardless of whether they were affected by the degenerative process. Magnesium is an important microelement, with approximately 60% stored in the bone, and the remaining 40% in the soft tissue [33]. The importance of this element in the diseases of the musculoskeletal system was ignored until a significant osteogenic effect of magnesium was noted. In humans, the concentration of magnesium in the serum has been found to be inversely related to the advancement of degenerative changes in the knee joint [34,35]. Feeney et al. showed that magnesium ions are an important factor regulating the circadian rhythm (cycle), which is a physiological, internal “clock” process regulating the rhythm of the day–night that repeats itself every day. It should be noted that magnesium is a cofactor of approximately 300 Adenosine 5′-triphosphate (ATP)-dependent enzymatic activities and a calcium channel antagonist, which makes it necessary for neuromuscular transmission. Thus, magnesium is involved in the biosynthesis of deoxyribonucleic acid (DNA) and ribonucleic acid (RNA), and proteins. Moreover, the magnesium released from the intracellular environment acts as a second messenger. Thus, this element plays an important role in the regulation of cellular and biological processes, as well as the induction or inhibition of signaling cascades, and its secretion depends on the time of day or night. This underlines its key role in the degeneration and repair processes in IVD [36]. It has been proven that magnesium deficiency in rats is responsible for the induction of inflammatory processes with the influx of macrophages and monocytes, the secretion of pro-inflammatory cytokines, and an increased concentration of free radicals. In such conditions, it is observed, inter alia, that there is an activation of the tumor necrosis factor alpha-dependent signaling pathway and the secretion of further inflammatory mediators along the signaling cascade. Therefore, the higher concentration of magnesium in the degenerated intervertebral discs, compared to the healthy intervertebral discs, may have arisen from the fact that the intervertebral discs affected by degenerative changes were obtained from human cadavers, which could reduce the concentration [37].

### 4.3. Potassium

Subsequently, we noted a statistically significant reduction in potassium concentration in the degenerated intervertebral discs, accompanied by a negligible, insignificant decrease in sodium concentration. Potassium is the main intracellular cation of the organism, which creates the resting and functional potential of the nerve cells, and is responsible for maintaining the water and acid–base balance. The disturbances in the concentrations of potassium and sodium ions are associated with the abnormal transport of potassium ions between the three spaces: cellular; extracellular; and intracellular [38]. The decrease in the potassium concentration noted by us may be the result of dehydration of the intervertebral disc, which is characteristic of degenerative diseases. The research shows that the outflow of potassium from the intracellular space with the simultaneous influx of calcium ions increases the secretion of reactive oxygen species (ROS), and induces and then progresses the inflammation and degrades the mitochondria [39]. It is interesting that, in the IVD obtained from patients reporting the severity of pain on the VAS scale, a statistically significant reduction in the concentration of potassium ions was observed, compared to the IVD obtained from patients reporting their pain level on the VAS scale (Student’s *t*-test, *p* < 0.05). First, this may be due to the disproportion between the size of the two groups, as well as the fact that both of the patients who reported a pain intensity level of 2 on the Pfirrmann scale also displayed the highest grade of radiological IVD degeneration. Thus, this seems to be supported by the fact that the VAS scale is very subjective, and it is possible that these two patients were taking painkillers regularly, which reduced their feelings of pain.

### 4.4. Iron

An element that plays an important role in the degenerative process is iron. Our study indicated a higher concentration of iron in the intervertebral discs affected by the process, compared to the controls. Although the changes in the iron concentration were not significant in the comparison between the study and control groups, the *p*-value for the comparison indicates that iron may be the element whose concentration profile most significantly changes in the intervertebral disc degeneration. Furthermore, when we considered the advancement of radiological changes, according to the Pfirrmann scale, in the statistical analysis, this speculation was supported. Iron is a cofactor of 25-hydroxycalciferol hydroxylase and participates in the hydroxylation of lysine and proline residues during collagen biosynthesis. Iron accumulation increases in post-mitotic tissues and increases with age [40].

Moreover, the observations of Nganvongpanit et al. showed a higher iron concentration in the synovial fluid in the degenerated knee joints. The decrease in the iron concentration, along with the advancement of the degenerative changes, may be one of the reasons for the loss of flexibility in the intervertebral disc, due to the reduction in the biosynthesis of collagen fibers and elastin, for which the iron ions are necessary [41].

### 4.5. Calcium

The last element for which we found significant changes in its concentration was calcium.

Karamouzian et al. were one of the first groups to demonstrate the more frequent occurrence of calcium salt deposits in the degenerated human intervertebral discs, compared to healthy ones (54.4% vs. 6.7%) [42]. Another important report confirming the presence of calcium deposits in intervertebral discs is based on a study by Gruber et al. These authors analyzed 208 human intervertebral discs of the lumbosacral spine, obtained during lumbar discectomy procedures. Deposits of calcium salts were visualized in 14.7% of the analyzed intervertebral discs [43], which is a much smaller percentage of cases than in the study by Karamouzian et al. (54.4%) [42]. In turn, Pytel et al., in a retrospective study, analyzed almost a thousand degenerated intervertebral discs obtained from all sections of the human spine during surgical procedures and showed the presence of calcium deposits in only 2.8% of the operated patients. Interestingly, in the degenerated intervertebral discs of the lumbosacral spine, compared to other sections, the incidence of calcification was slightly higher, amounting to 3.5% [44].

Additionally, the observations of Lee et al. are interesting; they indicated the presence of calcium deposits in only the intervertebral discs of the patients suffering from intervertebral disc degeneration. On the contrary, the occurrence of the calcium deposits in the intervertebral discs was completely underestimated in the patients suffering from lateral curvature of the spine [45].

Mass spectroscopy has so far only been used to assess the content of selected micronutrients in human intervertebral discs. For this reason, the method that we utilized allowed for the quantitative assessment of whole calcium in the intervertebral discs, and this is the first analysis of this type [45,46,47].

Stigen et al. conducted a study on a group of 25 dachshunds diagnosed with intervertebral disc degeneration, on which computed tomography was performed, as well as a histopathological examination of the removed degenerated intervertebral discs. These researchers indicated that, even in cases where the neuroimaging examination did not indicate any signs of intervertebral disc calcification, the presence of calcium deposits was found in the histopathological examination [46]. On the other hand, Grant et al. conducted interesting studies assessing the concentration of calcium in the degenerative intervertebral discs of the lumbosacral spine, with measurements taken up to 12 h after the patient’s death was confirmed. They indicated that the concentration of calcium was significantly higher in degenerating intervertebral discs with a higher advancement degree of degeneration, assessed according to the Thompson scale, which is consistent with our own results. The factor limiting the credibility of these studies was the fact that the study group consisted of only eight patients. These researchers also drew attention to the fact that the accumulation of calcium deposits significantly deteriorated the cellular metabolism and the availability of nutrients within the degenerated intervertebral disc. Illien-Jünger et al. conducted a study using five samples of the degenerated intervertebral discs of the lumbosacral spine, with the aim of assessing the incidence of calcium deposits, depending on the degree of degeneration of the spine, as determined using the Thompson scale [47]. Based on von Koss immunohistochemical staining, the regions of the so-called ectopic calcification within the studied intervertebral discs were visualized. The authors indicated that ectopic calcification may play a significant role in the accelerated degeneration of the intervertebral disc, inducing the initiation of structural defects [48]. Therefore, special attention should be given to the fact that a sine qua non condition for counteracting the progress of human IVDD of the lumbosacral spine should be lowering the calcium concentration to the values observed under physiological conditions [47].

Additionally, the observations of Dolor et al. confirmed that access reduction and impeded transport of nutrients to the intervertebral discs may be one of the significant causes of the biological failure of endo-disc therapies [49].

On the other hand, Chanchairujira et al., in a group of 3568 intervertebral discs obtained from deceased patients, with an average age of 67, using high-contrast radiography, showed the presence of calcification in only 469 intervertebral discs (13% of cases). The presence of calcium deposits in a relatively small group may result from the random selection of the group, in which patients without degenerative spine disease were also included [50]. For comparison, studies conducted by Feinberg et al. [43], using a similar methodology, showed the presence of calcium deposits in 34.5% of the analyzed patients [51]. In another study by Shan et al., which consisted of 73 patients with back pain, the presence of calcification in degenerated intervertebral discs was indicated in 94.1% of analyzed cases [52].

Nevertheless, it should be remembered that the calcification process of the intervertebral discs is not influenced by only the patient’s age [43]. In 1924, Lam et al. indicated the presence of calcium deposits in the intervertebral discs of small children, which was the case in only six of them [53]. More recently, Du et al. presented the story of a 6-year-old boy with calcifications of the intervertebral discs of the cervical spine, in which a significant clinical improvement was achieved after non-surgical treatment was conducted [54].

### 4.6. Relationship between Concentrations of Microelement in Healthy IVD and IVDD

In turn, the demonstrated statistically significant relationships between the concentrations of the individual elements in degenerated and healthy intervertebral discs suggest a striving towards a state of homeostasis by the external and internal factors, which occur in and affect the intervertebral discs. Indeed, the strong, positive correlations between some elements in degenerative IVD may suggest two parallel processes: degeneration and IVD regeneration. All of the correlations found in the IVDs of the study group concerned zinc and another microelement (lead, phosphorus, and calcium). Zinc is a cofactor of many of the enzymes involved in the degeneration of IVD, including matrix metalloproteinase (MMP), involved in the synthesis and degeneration of the extracellular matrix. Therefore, it is possible that the described relationships point to an attempt to achieve homeostasis in degenerated IVDs [55].

On the other hand, in IVDs obtained from the control group, a positive correlation was found between the concentrations of magnesium and sodium, and of calcium and ferrum. These correlations suggest a sufficient supply of nutrients to the IVDs taken from the control group, despite the fact that they were patients who underwent forensic section, which translates into high activity of DNA- and RNA-synthesis processes, probably also encoded by protein genes. These ions are cofactors of the enzymes involved in these processes; and the sodium ions, being a component of the sodium–potassium pump, ensure an adequate influx of nutrients to IVDs [26,56,57].

### 4.7. Strengths and Limitations

Of course, our study has both strengths and weaknesses. The strengths of the study include the fact that the assessment of pain symptoms was always performed in the morning by the same neurosurgery specialist, and the analysis of the MRI images was assessed by the same two intendent specialists in the field of neurosurgery, who then qualified the patients for surgery. Additionally, the presented results are among the few in which the concentrations of such a large number of elements in degenerated and healthy intervertebral discs were determined. It should also be emphasized that the research was conducted on a relatively large number of patients with intervertebral disc degeneration, and the obtained results were compared with the concentrations of the microelements in the healthy intervertebral discs. Nevertheless, it should also be remembered that this study is a single-center study with a disproportion between the sizes of the study and control groups. Therefore, more research is required. Another limiting factor in the conducted study is the method of obtaining samples from the control group. The material of the control group was collected posthumously, and the qualification for this group was based on an interview with the family of the deceased, who declared that they did not suffer and did not receive treatment for an IVD degenerative disease, of the L/S section of the spine. Nevertheless, it should be noted that the IVD taken from the control group was assessed histopathologically via H&E staining. It is unlikely that the post-mortem collection less than 48 h from the time of death could have a significant impact on the concentrations of the assessed micronutrients in IVD, especially as hypothermia is an important protective factor against degradation, e.g., of tissues, nucleic acid extracts, etc. [58,59]. On the other hand, of course, it should be borne in mind that the death of an organism stops the biological processes, which of course has a significant impact on the structures of the tissues and organs and the concentrations of the biochemical parameters or inflammatory mediators in the long run [60]. However, even in the normal IVDs, due to the low vascularization and innervation of the normal IVDs, the metabolism is largely anaerobic, and the main factor contributing to the death of the IVD cells is its progressive degeneration [61,62]. Nevertheless, there is no other method of obtaining the IVDs of correct human material besides during a post-mortem autopsy.

### 4.8. Future Perspectives

Furthermore, a valuable supplement to these obtained results would be determining the concentrations of these elements in the serum of people with IVD as well as healthy volunteers, in addition to searching for potential correlations between the concentrations of elements in the intervertebral discs and serum, which would probably increase the applicational potential of our study. Thus, taking into account the observations of Zhao et. al., who analyzed the correlations between the concentrations of calcium, phosphorus, potassium, sodium, and magnesium, and trace elements, such as zinc, iron, copper, and selenium, in the IVD and the serum of the patients with IVD degenerative disease of the L/S section of the spine, our attempt to determine the correlation should, in the first stage, concern the assessment of the calcium concentration, and then the subsequent elements. These authors noted that only for calcium was the relationship statistically significant (*r* = −0.332; *p* < 0.01), indicating that it may be a useful biochemical marker for the progression of the IVD degenerative disease of the L/S section of the spine [63].

## 5. Conclusions

Based on the conducted analysis, it can be concluded that the following elements are the most closely related to the degenerative process of the intervertebral disc of the lumbosacral spine: copper; potassium; calcium; and iron. It has been indicated that, above all, the concentrations of the copper and iron depend on the advancement of the radiological changes, according to the Pfirrmann scale; however, no influence on the pain intensity depending on the concentration of the assessed elements was found. The most significant relationships, found in the degenerated intervertebral discs, were between the following individual elements: Cu/Fe; Cu/Mg; Fe/P; Fe/Ca; Pb/Zn; Pb/P; Zn/Fe; Zn/K; Zn/Ca; Fe/Na. These were confirmed by the attempts to maintain homeostasis by the intervertebral disc during degeneration. Comparing our results with the observations of others, the most reasonable seems to be the determination of total calcium in IVD and the serum of patients suffering from degenerative IVD, in the lumbosacral section of the spine.

## Figures and Tables

**Table 1:** Inclusion and exclusion criteria for the study group.

Inclusion Criteria	Exclusion Criteria
Over 18 years of age	Under 18 years of age
Lumbosacral spine-isolated interverbal disc degeneration of a prolapse/extrusion char-acter determined through MRI	Degeneration of IVD of the lumbosacral spine of a protrusion or sequestration char-acter determined through MRI
Discogenic pain and/or symptomatic sciati-ca without improvement after non-surgical treatment for at least 6 weeks	Previous surgical procedures due to degen-eration of IVD of the lumbosacral spine
No other coexisting pathologies of the spine	Inflammatory and autoimmune diseases
Disease duration of no longer than 12 weeks	Condition after spine injury
	Dementia/mental disorders
	Polyneuropathy
	Pregnancy
	Coexisting diseases, including metabolic diseases
	Neoplasms: metastatic tumor in the spine; lymphoma; leukemia; spinal cord tumors; retroperitoneal tumors; primary shaft tumors
	Inflammatory diseases: inflammation of the bone elements of the spine
	Osteoporosis
	Duration of disease exceeding 12 weeks and no shorter than 6 weeks

**Table 2:** Inclusion and exclusion criteria for the control group.

Inclusion Criteria	Exclusion Criteria
Up to 45 years of age	Over 45 years of age
No signs of degeneration in the collected material in microscopic examination	Features of degeneration in the collected material in microscopic examination
No information about neoplastic diseases in disease history	In disease history, information about spinal diseases
Lack of information on inflammatory diseases: inflammation of the bone elements of the spine (osteomyelitis); inflammation of the intervertebral disc; epidural empyema; shingles; arthritis; inflammatory infiltrates of the rectum; Scheuermann’s disease; Paget’s disease	In disease history, information about neoplastic diseases
	Information on inflammatory diseases

**Table 3:** Concentrations of selected elements in healthy and degenerated intervertebral discs.

Microelement	Healthy Intervertebral Disc	Degenerated Intervertebral Disc	*p*-Value (Student’s *t*-Test)
Cu (mg/kg^−1^ dry matter)	5.90 ± 1.51	1.74 ± 1.31	0.000
Fe (mg/kg^−1^ dry matter)	104.57 ± 16.84	141.06 ± 58.22	0.069
Mn (mg/kg^−1^ dry matter)	0.20 ± 0.16	0.35 ± 0.52	0.407
Pb (mg/kg^−1^ dry matter)	0.81 ± 0.32	1.11 ± 0.76	0.243
Zn (mg/kg^−1^ dry matter)	21.76 ± 6.91	31.47 ± 18.42	0.127
Na (mg/kg^−1^ dry matter)	1.43 ± 0.20	1.42 ± 0.46	0.932
Mg (mg/kg^−1^ dry matter)	0.03 ± 0.01	0.26 ± 0.29	0.020
K (mg/kg^−1^ dry matter)	0.30 ± 0.11	0.14 ± 0.062	0.000
P (mg/kg^−1^ dry matter)	0.11 ± 0.02	0.79 ± 1.15	0.058
Ca (mg/kg^−1^ dry matter)	0.13 ± 0.03	1.93 ± 2.74	0.000

mean ± standard deviation; Cu, copper; Fe, iron; Mn, manganese; Pb, lead; Zn, zinc; Na, sodium; Mg, magnesium; K, potassium; P, phosphorus; Ca, calcium.

**Table 4:** Concentrations of selected elements in women and men in the study and control groups.

Microelement	Healthy Intervertebral Disc			Degenerated Intervertebral Disc		
	Female	Male	*p*-Value (Student’s *t*-Test)	Female	Male	*p*-Value (Student’s *t*-Test)
Cu (mg/kg^−1^ dry matter)	4.59 ± 1.54	6.12 ± 0.86	0.29	2.22 ± 2.48	1.71 ± 1.28	0.20
Fe (mg/kg^−1^ dry matter)	92.20 ± 24.32	109.45 ± 17.61	0.40	136.52 ± 56.81	141.43 ± 59.02	0.44
Mn (mg/kg^−1^ dry matter)	0.39 ± 0.33	0.15 ± 0.03	0.24	0.41 ± 0.68	0.29 ± 0.35	0.38
Pb (mg/kg^−1^ dry matter)	0.92 ± 0.45	0.66 ± 0.09	0.27	1.18 ± 0.89	1.10 ± 0.065	0.48
Zn (mg/kg^−1^ dry matter)	22.53 ± 8.93	14.49 ± 0.98	0.40	31.86 ± 21.23	31.62 ± 16.33	0.42
Na (mg/kg^−1^ dry matter)	1.63 ± 0.21	1.37 ± 0.18	0.29	1.50 ± 0.48	1.33 ± 0.42	0.44
Mg (mg/kg^−1^ dry matter)	0.031 ± 0.007	0.03 ± 0.006	0.46	0.24 ± 0.32	0.26 ± 0.26	0.26
K (mg/kg^−1^ dry matter)	0.33 ± 0.15	0.19 ± 0.03	0.29	0.14 ± 0.06	0.14 ± 0.07	0.41
P (mg/kg^−1^ dry matter)	0.04 ± 0.03	0.03 ± 0.01	0.39	0.92 ± 1.51	0.72 ± 0.81	0.22
Ca (mg/kg^−1^ dry matter)	0.12 ± 0.01	0.15 ± 0.02	0.41	2.19 ± 3.65	1.82 ± 1.93	0.38

mean ± standard deviation; Cu, copper; Fe, iron; Mn, manganese; Pb, lead; Zn, zinc; Na, sodium; Mg, magnesium; K, potassium; P, phosphorus; Ca, calcium.

**Table 5:** Concentrations of selected elements in the study and control groups depending on BMI value.

Microelement	Healthy Intervertebral Disc			Degenerated Intervertebral Disc			*p* < 0.05 (ANOVA Results) ^A,B^	*p* < 0.05 (Student’s *t*-Test) ^C,D,E^
	Normal Weight	Overweight	Obesity	Normal Weight	Overweight	Obesity		
Cu (mg/kg^−1^ dry matter)	4.99 ± 1.52	5.01 ± 1.76	5.03 ± 0.2.01	3.45 ± 1.72	4.18 ± 1.28	4.09 ± 2.11	*p* = 0.789 *p* = 0.654	*p* = 0.067 *p* = 0.051 *p* = 0.047
Fe (mg/kg^−1^ dry matter)	91.98 ± 21.22	93.412 ± 15.111	92.98 ± 15.11	126.52 ± 44.12	125.43 ± 51.01	228.52 ± 41.91	*p* = 0.754 *p* = 0.651	*p* = 0.021 *p* = 0.0004 *p* < 0.00001
Mn (mg/kg^−1^ dry matter)	0.29 ± 0.12	0.28 ± 0.03	0.30 ± 0.03	0.52 ± 0.12	0.549 ± 0.54	0.50 ± 0.63	*p* = 0.678 *p* = 0.734	*p* = 0.071 *p* = 0.041 *p* = 0.043
Pb (mg/kg^−1^ dry matter)	0.91 ± 0.41	0.89 ± 0.10	0.85 ± 0.08	1.36 ± 0.98	1.20 ± 0.065	1.34 ± 0.87	*p* = 0.632 *p* = 0.598	*p* = 0.034 *p* = 0.023 *p* = 0.021
Zn (mg/kg^−1^ dry matter)	21.53 ± 7.52	22.01 ± 0.34	22.02 ± 0.12	31.12 ± 21.13	30.82 ± 14.16	32.03 ± 19.99	*p* = 0.436 *p* = 0.513	*p* = 0.031 *p* = 0.012 *p* = 0.011
Na (mg/kg^−1^ dry matter)	1.73 ± 0.12	1.72 ± 0.14	1.70 ± 0.13	1.13 ± 0.39	1.10 ± 0.41	1.18 ± 0.49	*p* = 0.546 *p* = 0.567	*p* = 0.012 *p* = 0.014 *p* = 0.039
Mg (mg/kg^−1^ dry matter)	0.04 ± 0.007	0.03 ± 0.007	0.04 ± 0.001	0.03 ± 0.12	0.06 ± 0.31	0.03 ± 0.29	*p* = 0.671 *p* = 0.654	*p* = 0.459 *p* = 0.231 *p* = 0.762
K (mg/kg^−1^ dry matter)	0.43 ± 0.19	0.45 ± 0.18	0.47 ± 0.10	0.21 ± 0.03	0.19 ± 0.06	0.22 ± 0.04	*p* = 0.681 *p* = 0.661	*p* = 0.042 *p* = 0.012 *p* = 0.0512
P (mg/kg^−1^ dry matter)	0.03 ± 0.02	0.03 ± 0.01	0.03 ± 0.02	0.76 ± 1.22	0.72 ± 0.79	0.75 ± 1.44	*p* = 0.763 *p* = 0.719	*p* < 0.00001 *p* < 0.0001 *p* < 0.00001
Ca (mg/kg^−1^ dry matter)	0.15 ± 0.01	0.14 ± 0.02	0.14 ± 0.01	1.12 ± 2.01	1.23 ± 2.13	1.26 ± 1.82	*p* = 0.897 *p* = 0.759	*p* < 0.00001 *p* < 0.0001 *p* < 0.00001

mean ± standard deviation; Cu, copper; Fe, iron; Mn, manganese; Pb, lead; Zn, zinc; Na, sodium; Mg, magnesium; K, potassium; P, phosphorus; Ca, calcium; BMI, Body Mass Index; A—statistically significant differences between concentrations of microelements in healthy IVD depending on BMI (one-way ANOVA test; *p* > 0.05); B—statistically significant differences between concentrations of microelements in degenerative IVD depending on BMI (one-way ANOVA test; *p* > 0.05); C—statistically significant differences between microelements in healthy and degenerative IVD in patients with normal weight (Student’s *t*-test; *p* < 0.05); D—statistically significant differences between microelements in healthy and degenerative IVD in patients with normal weight (Student’s *t*-test; *p* < 0.05); E—statistically significant differences between microelements in healthy and degenerative IVD in patients with normal weight (Student’s *t*-test; *p* < 0.05).

**Table 6:** Concentrations of selected elements in degenerated intervertebral discs, depending on pain intensity.

Microelement (mg/kg^−1^ Dry Matter)		Cu	Fe	Mn	Pb	Zn	Na	Mg	K	P	Ca
VAS	*p* < 0.05	*p* = 0.207	*p* = 0.960	*p* = 0.374	*p* = 0.431	*p* = 0.154	*p* = 0.248	*p* = 0.871	*p* = 0.678	*p* = 0.574	*p* = 0.502
3		3.01 ± 2.53	132.00 ± 76.08	0.15 ± 0.01	0.60 ± 0.01	26.20 ± 10.20	1.18 ± 0.74	0.03 ± 0.01	0.0750 ± 0.06	0.05 ± 0.04	0.25 ± 0.08
4		2.10 ± 1.50	158.45 ± 68.55	0.20 ± 0.06	0.73 ± 0.26	19.73 ± 7,58	1.64 ± 0.01	0.28 ± 0.13	0.12 ± 0.03	0.22 ± 0.15	0.58 ± 0.33
5		1.48 ± 1.11	160.56 ± 70.01	0.53 ± 0.53	1.75 ± 1.30	54.18 ± 34.90	1.22 ± 0.35	0.25 ± 0.19	0.18 ± 0.11	1.68 ± 2.42	4.15 ± 6.29
6		1.22 ± 0.83	133.69 ± 55.37	0.32 ± 0.39	1.06 ± 0.69	29.72 ± 16.14	1.52 ± 0.36	0.31 ± 0.36	0.13 ± 0.03	1.08 ± 1.20	2.53 ± 2.61
7		2.060 ± 1.21	136.53 ± 82.74	1.11 ± 1.66	1.22 ± 0.53	39.15 ± 17.51	1.24 ± 0.73	0.12 ± 0.09	0.17 ± 0.04	0.72 ± 0.97	2.54 ± 1.80
8		2.31 ± 1.57	149.49 ± 63.11	0.33 ± 0.37	0.98 ± 0.58	31.76 ± 12.93	1.59 ± 0.47	0.33 ± 0.37	0.14 ± 0.04	0.66 ± 0.70	1.64 ± 1.63
9		2.67 ± 1.72	138.30 ± 59.31	0.15 ± 0.01	1.75 ± 1.28	29.03 ± 11.14	1.28 ± 0.58	0.14 ± 0.18	0.12 ± 0.02	0.4167 ± 0.33	0.57 ± 0.09
10		0.870000 ± 0.33	129.9750 ± 49.05	0.150000 ± 0.01	0.887500 ± 0.58	20.38000 ± 11.73	1.18 ± 0.55	0.27 ± 0.22	0.11 ± 0.03	0.440 ± 0.62	0.94 ± 1.15

mean ± standard deviation; Cu, copper; Fe, iron; Mn, manganese; Pb, lead; Zn, zinc; Na, sodium; Mg, magnesium; K, potassium; P, phosphorus; Ca, calcium; VAS, Visual Analogue Scale.

**Table 7:** Concentrations of selected elements in degenerated intervertebral discs, depending on the degree of radiological degeneration, according to the Pfirrmann scale.

Microelement (mg/kg^−1^ Dry Matter)		Cu	Fe	Mn	Pb	Zn	Na	Mg	K	P	Ca
Pfirrmann Scale	*p* < 0.05	*p* = 0.014	*p* = 0.003	*p* = 0.461	*p* = 0.488	*p* = 0.397	*p* = 0.633	*p* = 0.989	*p* = 0.292	*p* = 0.380	*p* = 0.348
2		3.71 ± 1.79	223.90 ± 21.37	0.17 ± 0.04	1.50 ± 1.26	48.71 ± 33.96	1.47 ± 0.31	0.08 ± 0.06	0.17 ± 0.07	1.65 ± 2.52	4.29 ± 6.41
3		2.31 ± 1.16	126.72 ± 52.19	0.71 ± 0.99	1.30 ± 0.82	30.98 ± 7.81	1.30 ± 0.64	0.08 ± 0.07	0.11 ± 0.02	0.42 ± 0.39	1.04 ± 0.85
4		1.56 ± 0.96	109.29 ± 58.21	0.33 ± 0.37	1.06 ± 0.54	40.02 ± 16.29	1.27 ± 0.47	0.08 ± 0.07	0.16 ± 0.09	0.72 ± 0.92	2.08 ± 2.22
5		1.12 ± 0.74	111.70 ± 44.24	0.43 ± 0.48	1.88 ± 1.04	36.41 ± 14.35	0.98 ± 0.38	0.09 ± 0.05	0.20 ± 0.13	1.11 ± 1.27	2.93 ± 3.27

mean ± standard deviation; Cu, copper; Fe, iron; Mn, manganese; Pb, lead; Zn, zinc; Na, sodium; Mg, magnesium; K, potassium; P, phosphorus; Ca, calcium.

**Table 8:** Correlations between the individual concentrations of elements in degenerated intervertebral discs.

Microelement	Cu	Fe	Mn	Pb	Zn	Na	Mg	K	P	Ca
Cu	1.00	0.48 *	0.16	0.15	0.17	0.18	−0.31 *	−0.15	−0.10	−0.10
Fe	0.48 *	1.00	−0.12	0.26	0.30 *	0.49 *	0.10	0.13	0.32 *	0.30 *
Mn	0.16	−0.12	1.00	0.05	0.02	0.13	−0.07	−0.16	−0.07	−0.04
Pb	0.15	0.26	0.05	1.00	0.67 *	−0.24	−0.27	0.15	0.52 *	0.58 *
Zn	0.17	0.30 *	0.02	0.67 *	1.00	−0.26	−0.15	0.38 *	0.74 *	0.77 *
Na	0.18	0.49 *	0.13	−0.24	−0.26	1.00	0.26	−0.13	−0.10	−0.14
Mg	−0.31 *	0.10	−0.07	−0.27	−0.15	0.26	1.00	−0.11	0.29	0.18
K	−0.15	0.13	−0.16	0.15	0.38 *	−0.13	−0.11	1.00	0.16	0.20
P	−0.10	0.32 *	−0.07	0.52 *	0.74 *	−0.10	0.29	0.16	1.00	0.98 *
Ca	−0.10	0.30 *	−0.04	0.58 *	0.77 *	−0.14	0.18	0.20	0.98 *	1.00

* statistically significant correlation; Cu, copper; Fe, iron; Mn, manganese; Pb, lead; Zn, zinc; Na, sodium; Mg, magnesium; K, potassium; P, phosphorus; Ca, calcium.

**Table 9:** Correlations between the individual concentrations of elements in healthy intervertebral discs.

Microelement	Cu	Fe	Mn	Pb	Zn	Na	Mg	K	P	Ca
Cu	1.00	0.70 *	−0.06	0.68 *	0.03	−0.12	0.16	0.22	0.37	0.23
Fe	0.70 *	1.00	0.11	0.45	0.14	−0.38	−0.18	−0.18	0.26	−0.06
Mn	−0.06	0.11	1.00	0.49	0.38	0.65	0.46	0.46	0.42	−0.34
Pb	0.68 *	0.45	0.49	1.00	0.29	0.43	0.52	0.43	0.38	−0.02
Zn	0.03	0.14	0.38	0.29	1.00	0.41	0.47	0.64	0.79 *	0.12
Na	−0.12	−0.38	0.65	0.43	0.41	1.00	0.90 *	0.64	0.31	0.25
Mg	0.16	−0.18	0.46	0.52	0.47	0.90 *	1.00	0.64	0.44	0.41
K	0.22	−0.18	0.46	0.43	0.64	0.64	0.64	1.00	0.74 *	0.10
P	0.37	0.26	0.42	0.38	0.79 *	0.31	0.44	0.74 *	1.00	0.13
Ca	0.23	−0.06	−0.34	−0.02	0.12	0.25	0.41	0.10	0.13	1.00

* statistically significant correlation; Cu, copper; Fe, iron; Mn, manganese; Pb, lead; Zn, zinc; Na, sodium; Mg, magnesium; K, potassium; P, phosphorus; Ca, calcium.

## Data Availability

The data used to support the findings of this study are included in the article. The data will not be shared due to third-party rights and commercial confidentiality.
